# The prevalence of self-reported vision difficulty in economically disadvantaged regions of South Africa

**DOI:** 10.4102/ajod.v4i1.136

**Published:** 2015-06-25

**Authors:** Kovin S. Naidoo, Jyoti Jaggernath, Prasidh Ramson, Farai Chinanayi, Tom Zhuwau, Lene Øverland

**Affiliations:** 1 Brien Holden Vision Institute, Durban, South Africa; 2 African Vision Research Institute, University of KwaZulu-Natal, South Africa; 3 Vision Cooperative Research Centre, Sydney, Australia; 4 Orbis, Cape Town, South Africa; 5 Department of Rural Development and Land Reform, South Africa

## Abstract

**Background:**

Vision impairment, resulting in vision difficulties, is a leading cause of disability, and hence one of the key barriers for people to access education and employment, which may force them into poverty.

**Objectives:**

The objective of this study was to determine the prevalence of self-reported vision difficulties as an indicator of vision impairment in economically disadvantaged regions in South Africa, and to examine the relationship between self-reported vision difficulties and socio-economic markers of poverty, namely, income, education and health service needs.

**Methods:**

A cross-sectional study was conducted in economically disadvantaged districts to collect data from households on poverty and health, including vision difficulty. As visual acuity measurements were not conducted, the researchers used the term vision difficulty as an indicator of vision impairment. Data were collected from 27 districts (74 901 respondents). Logistic regression analysis and chi-square tests were used to determine bivariate relationships between variables and self-reported vision difficulty. Kernel density estimators were used for age, categorised by self-reported and not reported vision difficulty.

**Results:**

Prevalence of self-reported vision difficulty was 11.2% (95% CI, 8.7% – 13.7%). More women (12.7%) compared to men (9.5%) self-reported vision difficulty (*p* < 0.01). Self-reported vision difficulty was higher (14.2%) for respondents that do not spend any money. A statistically significant relationship was found between the highest level of education and self-reporting of vision difficulty; as completed highest level of education increased, self-reporting of vision difficulty became lower (*p* < 0.01). A significantly higher prevalence of self-reported vision difficulty was found in respondents who are employed (*p* < 0.01), 17% (95% CI: 12.8% – 21.1%).

**Conclusion:**

The evidence from this study suggests associations between socio-economic factors and vision difficulties that have a two-fold relationship (some factors such as education, and access to eye health services are associated with vision difficulty whilst vision difficulty may trap people in their current poverty or deepen their poverty status). The results are thus indicative of the need for further research in South Africa.

## Introduction

Vision impairment, causing vision difficulties, is the leading cause of disability, and hence one of the key barriers for people in South Africa to access education and the labour market, which may force them into poverty. The War on Poverty Campaign (WOPC), an initiative of the South African Government to provide services and support for the ‘poorest of the poor’ families in the country, has developed a large database of households that were identified as being economically disadvantaged. The WOPC was commissioned by the presidency as part of the government's Apex priorities, with the deputy president as the lead person. The campaign seeks to raise the profile of current poverty eradication strategies and reach out to more people in South Africa. In addition, the campaign attempts to ensure that civil society, business, non-government organisations, and community-based organisations join in the anti-poverty effort (Santiaguel n.d.). The WOPC resolved to define the poverty matrix in the country; develop a database of households living in poverty; identify and implement specific interventions related to these households; monitor progress in moving households out of poverty, and coordinate and align poverty eradication programmes to maximise impact and avoid wastage and duplication.

In line with the objectives of the WOPC, a strategic partnership was formed between three eye health institutions namely, Orbis, the Brien Holden Vision Institute, and the African Vision Research Institute (AVRI) and the Department of Social Development to investigate poverty and eye health in South Africa. As part of the WOPC, this research collaboration brought together specialists – the researchers and authors – in the field of eye health to investigate the prevalence of eye diseases in South Africa. This three-pronged collaboration also aims to identify, implement, and monitor eye health services that are desperately needed to improve sight. This article, however, only presents data that were collected from the WOPC initiative, which provided the researchers with a select sample of poor households that had been identified with self-reported vision difficulties. Other studies, as part of this collaboration, are currently being conducted. In previous studies a general population would have been sampled, prevalence of blindness and vision impairment determined by means of visual acuity measurements, and then correlated with employment, education, quality of life and other socio-economic indicators. In this study the researchers have, however, direct access to households that the WOPC identified as being in poverty. Data collected from the WOPC include information on health and vision difficulties. Thus, access to the WOPC database provided the researchers with an opportunity to determine the prevalence of self-reported vision difficulties in economically disadvantaged regions of South Africa.

The purpose of this article was, therefore, to explore and analyse demographic (age, gender, education), socio-economic (housing, employment, social and documentation) needs, self-reported vision difficulty data as an indicator of vision impairment, and eye health service needs that were extracted from the WOPC database. The article examines the relationship between self-reported vision difficulty and indicators such as income, education and eye health service needs which are often used as markers for poverty in resource limited communities.

In this study we sought subjective (self-reported) responses. As visual acuity measurements were not conducted, the researchers used the term vision difficulty as an indicator of vision impairment which is usually classified by the World Health Organisation (WHO n.d.) according to a measured visual acuity.

### Literature review

#### Poverty in South Africa

South Africa has a population of 51.8 million people (Statistics South Africa [StatsSA] 2011a) and a gross domestic product (GDP) per capita per annum of R80 780.10 ($6617.90) (World Bank 2015a). Approximately 22% of the population in South Africa is living on less than purchasing power parity (PPP) of $2.00 a day (World Bank 2015b), most of whom are black South Africans. In 1995, an estimated 58% of South Africa's population was categorised as being poor, calculated by using a national lower-bound poverty line of R2000.00 (R322.00 per person) per month (Özler 2007:499). The poverty head count in 2011 was 56.8% (StatsSA 2011b). The income Gini coefficient in South Africa stands at an alarming high 0.7 (Organisation for Economic Co-operation and Development [OECD] 2013), whilst Norway has the lowest Gini index of 25.6% as reported in 2012 (EuroMonitor International 2012). This suggests that probably nowhere else in the world are so many people more privileged in one country whilst others in the same country live close to or below the poverty line.

High rates of unemployment, poverty and inequality in South Africa can be traced to colonial exploitation and the apartheid system as well as policies that denied African people access to opportunities, including access to land, to run businesses, to own certain assets, to quality education and to live in areas that were well established and located (Office of the Presidency 2011). Whilst advances in areas such as electrification and access to education have increased equality of opportunities (World Bank 2012), the Gini coefficient for South Africa (0.66 in 2003 to 0.7 in 2008) (Leibbrandt *et al.* 2010:10) suggests that there has been no progress towards income equality, almost 20 years after the end of apartheid. Ethnicity accounts for a large part of income inequality (OECD 2013). However, data collected via the WOPC do not provide an analysis on income inequality in South Africa.

The *District Health Barometer* (2008/2009) indicated that 10 of the most deprived districts in South Africa in 2006 fell within three provinces, namely, KwaZulu-Natal, Eastern Cape and Limpopo which are predominantly rural with a large population size compared to other provinces in the country (Day *et al.* 2009). Households living on less than R800.00 ($106.00) per month ranged between 63% and 82% in the 10 districts. The *Afrobarometer poverty survey's Lived Poverty Index* (LPI) for 2012 indicates that poverty in South Africa has increased despite reported average economic growth of 3.6% between 2002 and 2012 (Dulani, Mattes & Logan 2013:12).

Poverty is much higher in rural areas, particularly in the former bantustans; however with the inward migration of people in search of work, deep poverty is also found in cities (Office of the President 2011:09). More than two-thirds (68.8%) of rural dwellers were estimated to be living in poverty in 2011, as compared with less than a third (30.9%) of residents in urban areas (StatsSA 2014:33). Furthermore, poverty amongst female-headed households and amongst children is also higher than average. Women are more impoverished than men in South Africa, with a headcount of 58.6% in 2011 as compared with 54.9% for men (StatsSA 2011b). Women and children experience higher vulnerability to poverty, with an increasing number of them finding themselves as heads of households, especially in rural areas (Lewis 2001). According to Lewis (2001:73) women's economic and social status in South Africa has been largely determined in relation to men. Overall, women tend to have less access to resources than men. In 2011, estimates showed that children constituted 37.6% of the total South African population and, alarmingly, almost half (46.6%) of all poor people in South Africa were children (StatsSA 2014:28).

Poverty is closely related to poor education and lack of employment. Almost 25% of the population is unemployed (StatsSA 2011b). Klasen (1997:51) suggests that the poor experience a lack of access to education, quality health care, basic infrastructure, transport, and productive resources. They are heavily indebted and depend largely on social grants, particularly social pensions and disability grants. In addition, those who are poor also have limited access to health services because of factors such as prohibitive transportation costs (Klasen 1997:51).

According to May, the nature of poverty in South Africa is changing as is evident in the rural-urban migration trend, whereby the urban population increased by 9.5 million, thereby increasing the number of urban poor by 4.7 million, whilst the number of rural poor declined by 770 000 (May 2010:02). Despite this, livelihoods in poor rural communities are characterised by asset poverty, lack of access to the resources for food production and high levels of monetisation and integration into the broader economy (Du Toit 2005:09–10). This leads to a high degree of cash-dependency, low levels of education and subsequent low employment levels and insecure unskilled jobs (Du Toit 2005:10).

## Vision impairment and blindness in South Africa

According to the South African National Council for the Blind (SANCB 2011) there are over 724 000 people in South Africa who may be experiencing vision impairment and thus vision difficulties. Vision impairment is defined as unable to see at 6 metres what a normal sighted person will see at 12 metres or vision worse than this (Dandona and Dandona 2006:04). The *South African Census 2001* (StatsSA 2001) data indicate that 2 255 982 South African individuals (5% of the total population) self-reported that they had some form of disability. The largest proportion of individuals (32%) indicated that they had vision disability, 30% a physically disability, 20% a hearing impairment, 16% were emotionally disabled, 12% an intellectual disability and 7% indicated that they had a communication disability. In the South African Census 2011, disability was defined as difficulties encountered in functioning as a result of body impairments or activity limitation, with or without the use of assistive devices (StatsSA 2011c). Less than 11% of the population reported having difficulties, with the largest proportion indicating that they had vision difficulties (11.1%). Other difficulties were reported as hearing (3.5%), communicating (1.6%), walking or climbing stairs (3.5%), remembering or concentrating (4.4%), self-care (3.4%). Spectacles and chronic medication (14% and 12% respectively) were reported (the total population surveyed) as more used compared to other assistive devices (StatsSA 2011a).

There are 400 000 people in South Africa who are blind, that is, unable to see at three metres what a normal sighted person sees at 60 metres or vision worse than this (Mercy Vision 2013:01). The leading causes of blindness are cataracts (66%) and glaucoma (14%), and the majority of people affected (80%) live in the rural areas (South African National Department of Health 2002). Cataracts account for 190 000 blind people in South Africa. It is further estimated that around 9000 children are blind and would benefit from eye care (International Finance Cooperation [IFC] 2007). Moreover, the South African Disability Alliance (SADA) report indicates that women make up 57% of the total number of blind and vision impaired people in South Africa (SADA Report n.d.:31). The SADA report further indicates that 97% of vision impaired South Africans are unemployed. The high percentage of unemployed people with vision impairment could be because of vision difficulties in carrying out work tasks that is dependent on good vision. About 23% are between the ages of 15 and 36 years, and only about 5% to 10% of the total blind population is Braille literate (SADA n.d.31).

### 

#### Eye care services in South Africa

The South African Government has restructured the health system to improve equity and access to primary health care after the first democratic elections in 1994. However, major inequities remain despite achieving democracy and new leadership. Across South Africa's nine provinces, there are significant variations in health status and access to health services (Lawn & Kinney 2009:02), including eye care services.

The overwhelming majority of South Africa's poor live in rural areas without access to quality eye care services. Current estimates indicate that there are 3408 optometrists registered with the Health Professions Council of South Africa (Health Professionals Council of South Africa [HPCSA] 2013). A study conducted by the Brien Holden Vision Institute showed that about 97% of optometrists are practising in the private sector in the various provinces of South Africa (Brien Holden Vision Institute 2006). There are approximately 325 ophthalmologists in South Africa, of whom about 70 work in the public sector serving 80% of the population (Lecuona & Cook 2011). Additional barriers to eye care services for poor people include the cost of travel to and from hospital as well as the exorbitant cost of accessing eye care from the private sector. This has serious implication for the ability of the majority of the population to access eye care. There are limited services available to the general majority, such as screening and basic treatment at a primary health care clinic, despite South Africa being one of the most stable countries in the region.

Furthermore, ophthalmology and optometry services are expensive and, until just a few years ago, were almost exclusively accessible only to those living in urban areas through the private sector (Rawlings 2011:03). In addition to the lack of adequate and accessible services, the expensive nature of spectacles makes them cost prohibitive to the poor. The inaccessibility of spectacles is an equally large problem because of the unavailability of spectacle technicians, manufacturers, and vendors in the rural areas (Rawlings 2011:03).

Theoretical evidence that poverty and poor eye health in developing countries is closely related has been succinctly captured in a literature review documented by Jaggernath *et al.* (2014). The evidence documented indicates that poor people have limited access to employment opportunities, basic services and health care and treatment that affect eye health and vision restoration (Gooding 2006:02; Naidoo 2007:417; Mitra, Posarac & Vick 2011:05–06). In developing countries such as South Africa, the state of eye health is disconcerting, despite the various interventions that have been subsidised to address the problem (Jaggernath *et al.* 2014:1856). Access to eye health services are difficult for people who are poor, and poor communities are challenged with barriers such as inadequate clinical services and finances, limited eye health knowledge and geographical barriers (Jaggernath *et al.* 2014:1853). Vision impairment and blindness is likely to impact on social and economic opportunities for affected individuals (Dandona & Dandona 2001:222; Ho & Schwab 2001:653) and can contribute to plunging individuals further into poverty. According to Jaggernath *et al*., people experiencing at least one marker of poverty, such as employment or income, are more likely to be prone to certain eye diseases, less likely to seek treatment, or timely treatment, for their eye condition or disease and are more likely to have poor outcomes, as a result of barriers they face following treatment (Jaggernath *et al.* 2014:1857). However, there has been little research conducted on the prevalence of vision difficulties related to vision impairment or blindness in South Africa and the associations between poverty and vision impairment indicators, such as vision difficulties experienced by individuals who are economically disadvantaged.

#### Aim and objectives of the study

In considering the literature consulted on poverty, vision impairment, blindness and eye health services in South Africa, the aim of this study was to determine the association between self-reported vision difficulty, a proxy indicator of vision impairment, and socio-economic status in economically disadvantaged districts in South Africa. Specific objectives include determining the prevalence of self-reported vision difficulty in the economically disadvantaged regions of South Africa and examining the relationship between self-reported vision difficulty and indicators such as income, education and health service needs which are often used as markers for poverty in resource limited communities.

## Research method and design

### Research setting and design

A cross-sectional study was conducted by the Department of Social Development in South African. The study was conducted in way of a census by eliciting data on poverty and health, including vision difficulties from all respondents within selected economically disadvantaged and not economically challenged wards.

Wards were allocated a poverty index and wards that were most economically challenged were identified through a prioritisation process using key indicators such as income, education level, and employment and mapping households in those wards to confirm their status as households in poverty. Twenty seven districts from all nine provinces in South Africa were found to be most economically challenged and 18 016 poverty-struck households that were randomly selected from these districts were profiled by the WOPC. Data were obtained from 74 901 respondents.

### Procedure

The WOPC collected household data by way of a census, using survey questionnaires. The preferred method of data collection was mobile phone questionnaires, because they were directly linked to the government's database and responses from participants are automatically stored on the database. Face-to-face interviews need to be manually entered into the government database, thus, when respondents had no access to mobile phones, face-to-face interviews were conducted. Data were obtained from 51.8% of households through mobile phone questionnaires and 48.2% of households by conducting face-to-face interviews. Questionnaires included sections for demographic data, socio-economic conditions and health and disability. Questions on health and disability included vision difficulty. The data were collected at the household level by trained enumerators. Information was provided by a household representative, either by the household head or any other member of the household who was over the age of 18 years. Following data collection, the questionnaire responses were captured by trained data entry technicians into an excel database. This database provided an opportunity to extract, analyse and disseminate information on poverty and self-reported vision difficulty in the economically disadvantaged districts in South Africa and investigate the prevalence of self-reported vision difficulty as well the relationship between vision difficulty and poverty, using indicators for poverty, such as income, education, and health service needs in resource limited communities. These indicators were selected as they formed questions in the WOPC questionnaire that related to the researchers’ objectives of correlating poverty markers with self-reported vision difficulties.

### Data analysis

Logistic regression analysis was conducted to determine the association between poverty indicators and vision difficulty. Chi-square tests were employed to determine bivariate relationships between each variable and the reported vision difficulty. Table 1 shows the operational definition of each variable. The results generated are subjective, that is, dependent on how the respondent rated himself or herself, or how the proxy rated another household member.

**TABLE 1:** Operational definitions of variables.

Data variable	Data explanation	Data type	Categories
Visual difficulty	Dependent: Difficulty in seeing even when using glasses	Categorical	0=No; 1=Yes; 99=Do not know*
	Independent variables	-	-
Age	Age at last birthday	Continuous	-
Gender	Sex of the respondent	Categorical	1=Male; 2=Female
Education	Highest education level successfully completed	Categorical	0=no formal school; 1=primary school; 2=high school; 3=tertiary education
Expenditure	Total household expenditure in the last month	Categorical	1=0; 2=1-199; 3=200-399; 4=400-799; 5=800-1199; 6=1200-1799; 7=1800-2499; 8=2500-4999; 9=50-9999; 10=100 or more; 11=Don't know; 12=Refused*
Heath care needs	Independent	Categorical	0=No; 1=Yes
Education needs	Independent	Categorical	0=No; 1=Yes
Documentation needs	Independent	Categorical	0=No; 1=Yes
Employment	Employment status	Categorical	0=No; 1=Yes
Province	Province of residence in South Africa	Categorical	0=No; 1=Yes
Household size	Total number of household members	Continuous	0=No; 1=Yes
Each option for occupation	-	Categorical	0=No; 1=Yes
Each option for business activities	-	Categorical	0=No; 1=Yes

*, a, do not know; b, refused or c, not stated were removed from the analysis but are presented in Tables.

The question on income in the WOPC questionnaire was scarcely reported; therefore, a limitation to the analysis was that respondents’ expenditure was utilised as a proxy to income. Importantly, there are three categories that do not state the exact expenditure: do not know, refused, and not stated. Non-responses were removed from the analysis.

Age distributions were compared between respondents who self-reported having vision difficulty even when wearing spectacles and those who did not have any vision difficulty. This was conducted by plotting kernel density estimators for age, categorised by self-reported and not reported vision difficulty.

Vision difficulty was examined in relation to the education and health care needs. Education needs include school feeding, fees and uniform, scholar transport, career guidance, access to bursaries, special educational needs, vocational skills development, further education and training (FET) and textbooks. Health service needs included the need for a road to health card (RTC), treatment or medication requirement, medical check-up, rehabilitation services, assistive devices, nutrition programmes, voluntary counselling and testing (VCT), immunisation and anthropometric measurements. In addition, documentation needs were combined to include identity document (ID), birth, marriage and death certificate, passport and residence permit.

Working was defined in the study as being formally employed and earning a salary. This question was administered to respondents who are above the age of 15 years and respondents who are not currently attending schools. In consideration to the possibility that some respondents may have multiple skills, respondents were given a multiple responses option for confirming their different skills. All tests were conducted at the 5% level of significance, taking into consideration the sample design.

## Results

The sample comprised of 55% women and 45% men. Respondents less than 15 years old comprised 32% of the sample, 32% were between the ages 15 and 34 years and 30% were aged 35 years and older. The ages were not stated by 6% of the respondents.

The prevalence of self-reported vision difficulty was 11.2% (95% CI: 8.7% – 13.7%) with 32.7% of these respondents wearing spectacles. Of the total sample 5.1% (95% CI: 3.78% – 6.36%) wore spectacles. In addition, 9.8% (95% CI: 7.29% – 12.30%) self-reported that their eye condition was permanent. A significant percentage of women (12.7%) compared to men 9.5% self-reported vision difficulty (*p* < 0.01).

The highest level of education was not reported by 3% of the respondents. Educational level categories included no formal schooling, primary schooling, secondary school and tertiary education. There were 14.7% of respondents who did not state an education category; they, however, self-reported vision difficulty and were removed from this particular analysis. There is a statistically significant relationship between highest level of education and self-reporting of vision difficulty (*p* < 0.01); as completed highest level of education increased, self-reporting of vision difficulty became lower. Respondents with at most primary schooling self-reported more vision difficulty compared to respondents with at least secondary schooling.

**FIGURE 1:**
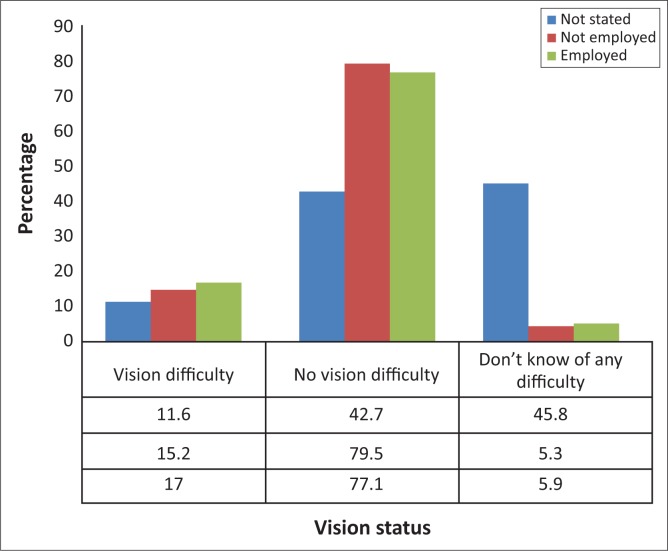
Employment and vision status.

Self-reported vision difficulty was higher for respondents who are dependent on others (14.2%) and respondents who spend R10 000 or more per month (15.9%). Respondents who were employed have a significantly higher prevalence of self-reported vision impairment than those who are not employed (*p* < 0.01), 17% (95% CI: 12.8% –21.1%), compared to 15.2% (95% CI: 11.3% – 19.0%) as depicted in Figure 1. Employed respondents were 1.15 (1.08 – 1.23) times more likely to self-report vision impairment compared to the unemployed respondents.

**FIGURE 2:**
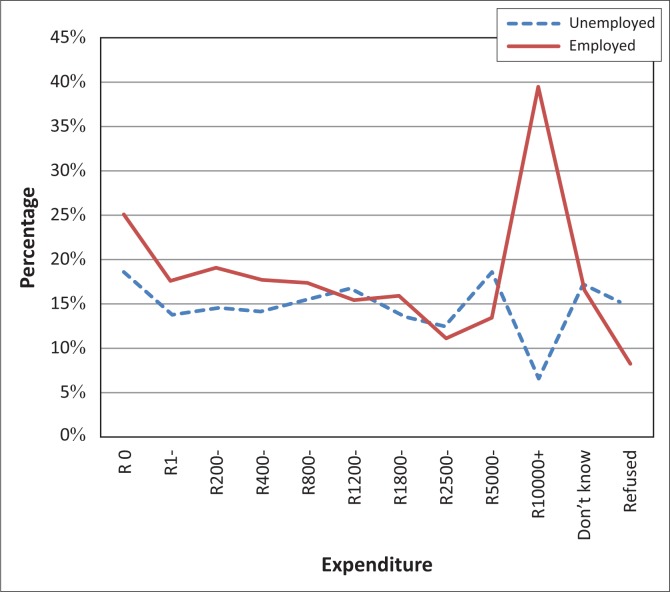
Self-reporting of vision difficulty by expenditure and employment status.

Expenditure was reported at household level and vision difficulty was self-reported at an individual level. However, it can be observed that self-reporting of vision difficulty for both the unemployed and the employed decreases as the household expenditure increases, save for household expenditure greater than R10 000 (Figure 2).

**FIGURE 3:**
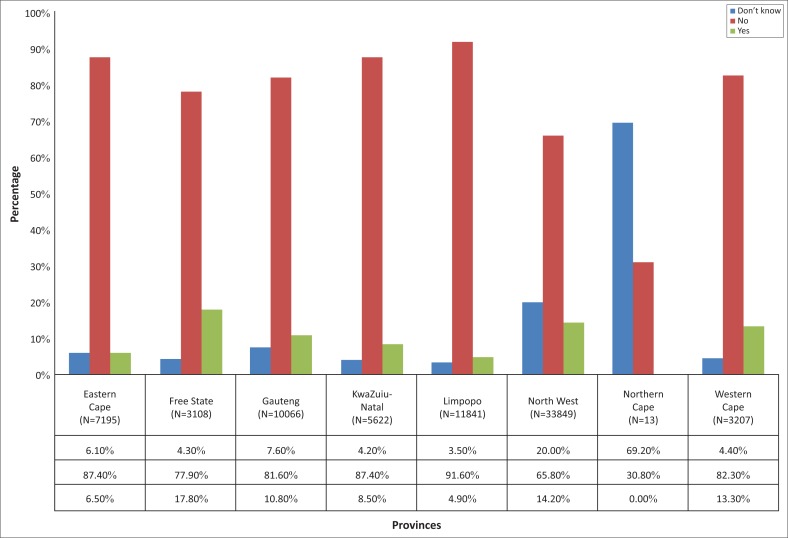
Self-reported vision difficulty in the nine provinces of South Africa.

Self-reported vision difficulty was significantly higher for the Free State, North West, Western Cape and Gauteng provinces with 17.8%, 14.2%, 13.3% and 10.8% respectively (Figure 3).

**FIGURE 4:**
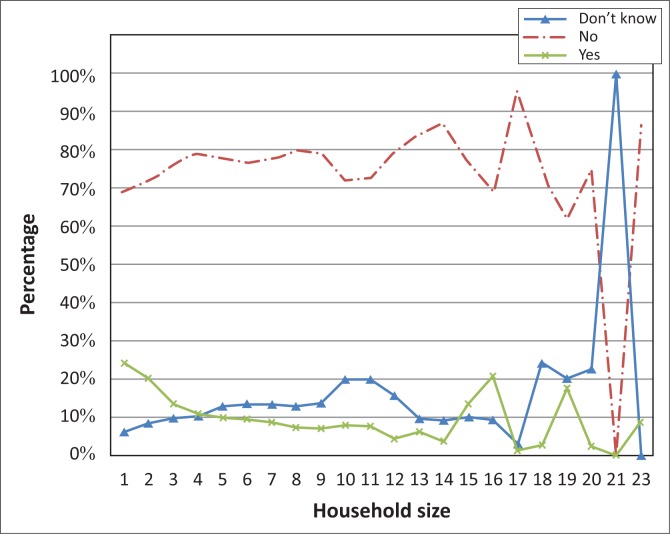
Self-reported vision difficulty by household size.

The highest number of occupants in a single household was 23 (figure 4). Self-reporting of vision difficulty is erratic for households with more than 12 occupants as a result of the small frequencies observed for these households. Respondents are 0.95 (95% CI: 0.94% – 0.97%) times less likely to self-report vision difficulty for each unit increase in household size (*p* < 0.01). Importantly, it was observed that one household representative reported on behalf of each household member; therefore, vision difficulty may not have been reported in bigger households.

The following occupations were significantly related to self-reported vision difficulty: painting (*p* = 0.01), bricklaying (*p* < 0.01), waiter or waitress (*p* = 0.04), home and community-based care giving (*p* < 0.01), welding (*p* < 0.01), carpentry (*p* = 0.03), electrical (*p* < 0.01), child care (*p* < 0.01) farming (*p* < 0.01) and sewing (*p* < 0.01). Table 2 shows that respondents involved in sewing are 2.1 times more likely to have vision difficulty than those without the skill. On the other hand respondents with electrical skills are 0.7 times less likely to self-report vision difficulty than those without the skill. However, there was no significant relationship between vision difficulty and security, plumbing, plastering or book keeping skills.

**TABLE 2:** Odds ratios for skills.

Skill	Oddsratio (95%CI)	*p* -value
Painting	1.2 (1.06–1.43)	(p = 0.01)
Bricklaying	1.3 (1.12–1.42)	(p < 0.01)
Waiter	0.8 (0.68–0.99)	(p = 0.041)
Security	0.9 (0.64–1.24)	(p = 0.493)
HCBC	1.4 (1.13–1.64)	(p < 0.01)
Welding	1.4 (1.25–1.66)	(p < 0.01)
Carpentry	1.2 (1.02–1.46)	(p = 0.029)
Electrical	0.7 (0.54–0.85)	(p < 0.01)
Plumbing	1.0 (0.76–1.33)	(p = 0.978)
Childcare	1.4 (1.15–1.62)	(p < 0.01)
Plastering	1.1 (0.83–1.34)	(p = 0.660)
Farming	1.7 (1.45–2.03)	(p < 0.01)
Sewing	2.1 (1.81–2.40)	(p < 0.01)
Bookkeeping	0.9 (0.61–1.35)	(p = 0.615)

**FIGURE 5:**
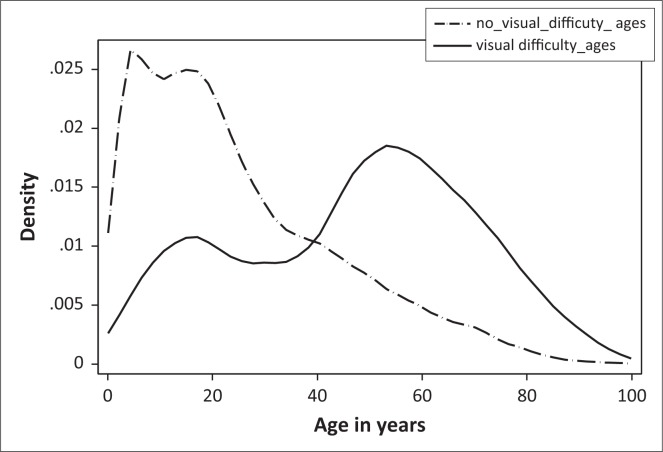
Comparisons between ages for those with self-reported vision difficulties and those without vision difficulties.

More respondents aged below 40 years reported that they have no vision difficulty in comparison to those who are above 40 (Figure 5). The density plot for individuals with self-reported vision difficulty showed a bimodal distribution with peaks at about 18 and 58 years. Therefore, relatively more people in these age groups self-reported vision difficulty even when wearing spectacles. When comparing the two age plots for those below 40 years, more individuals did not report vision difficulty whilst the converse is correct for individuals aged above 40 years. At the age of 40 years, the number is the same for people that self-reported vision difficulty and those who reported that they had no vision difficulty.

The distributions of the two functions using the Kolmogorov-Smirnov test for equality between distribution functions (figure 5) conclude that the two groups have different distribution functions (*p* < 0.01). In addition the mean ages for those self-reporting vision difficulty and those who are not self-reporting vision difficulty were, respectively, significantly different (*p* < 0.01).

In relation to business activities some households reported multiple responses for the types of business activities they were involved in; meaning that they are involved in several business activities. In addition, when a business activity is reported for a household, every individual in that household is reported to be engaged in that business activity regardless of who is taking part in the type of business. Considering this, removing minors from the analysis would be speculative. This is because most of these businesses are operated from home and minors are exposed to similar environments as adults. For example, a 10-year old child may assist in selling goods or services in their family spaza [tuck shop] after school. About 7% of the respondents reported that they were involved in the selling of goods and services on the streets. Other business activities mentioned involved hair salons, welding, panel beating, vehicle repairs, wedding planners, property owners and carpenters. The self-reported vision difficulty for the different categories ranged from a high of 20% (30 out of 150) for those in the wood or fuel business and a low of 8% for those transporting goods.

Health and education needs were highest amongst respondents, 20.7% and 19.6% respectively, whilst social and documentation needs were 9.7% and 9.3%, respectively. The results show that 21.8% of the respondents with health needs self-reported that they have vision difficulty, with 5.8% of the respondents with educational needs self-reporting vision difficulty.

There is a significant relationship between self-reporting of vision difficulty and education needs (*p* < 0.01) and/or health needs (*p* < 0.01). However, there was no significant relationship between self-reporting of vision difficulty and documents needs (*p* = 0.91). In addition, no significant relationship was found between self-reporting of vision difficulty and social needs (*p* = 0.20). The respondents with education needs were 0.42 (95% CI: 0.35% – 0.49%) times less likely to self-report vision difficulty than those who had no education needs (*p* < 0.01). The respondents with health needs were 3.04 (2.50 – 3.69) times more likely to self-report vision difficulty that those without health needs (*p* < 0.01).

### 

#### Ethical considerations

Ethical clearance for presenting data extracted from the WOPC database was received from the University of KwaZulu-Natal Humanities and Social Sciences Research Ethics Committee and the South African Department of Social Development.

#### Data protection

The primary research that informed this article was conducted by the government (Department of Social Development) in areas defined as economically disadvantaged in South Africa. Although the survey instrument used collected household members names and addresses, the data that were retrieved from the Department of Social Development were anonymous. Thus the responses received were not linked to any particular individual or households.

#### Trustworthiness

Secondary data were employed; therefore, particular information pertaining to questionnaire development and data collection process was carefully examined so as to inform the analysis methods and the limitations of the research results.

#### Reliability

This research employed a quantitative approach; therefore the meaning of reported numbers will remain consistent over time. The questionnaire used was a revised version of a preceding similar study after removing ambiguous sections. In addition, the sampling entailed the profiling of wards in South Africa, followed by conducting censuses in the selected wards which increased the sample size making the estimates accurate for the population.

#### Validity

Validity determines if the research truly measures what it is intended to measure (Joppe 2000). The questionnaire did not include leading questions that may skew the responses. Sections were constructed to elicit particular data needed to assess socioeconomic status. The question relating to vision difficulty was either ‘yes’ or ‘no’ without any follow-up questions, reducing conflicting responses. Vision difficulty was self-reported by the respondent without any incentive offered such as the provision for eye care.

## Discussion

The prevalence of self-reported vision impairment or blindness found in this study (11.2%) is lower than the prevalence found in other prevalence studies (Bucher & Ijesselmuiden 1988; Cook, Night & Crofton-Briggs 1993). Previous studies in South Africa were epidemiological evaluations which depended on objective measures such as visual acuity.

Household size was a significant factor in the self-reported vision impairment. This could be the result of lesser resources or that limited resources have to be prioritised and vision is, thus, not high on the agenda when such choices have to be made. However, there is no definitive data to conclude this and further investigation in this regard will be useful.

When resources are limited, families prioritise certain expenses or services with women and girls being considered less of a priority (Courtright & Lewallen 2007:68, 2009:18), especially if women are unemployed. Furthermore, if they are not working, they are not in the position of making decisions on eye health expenditure. This may explain the higher level of vision impairment self-reported by women in comparison to men. There is no clear trend in the self-reporting of vision impairment and education. Whilst the percentage of respondents (12.3%) with primary schooling self-reporting vision impairment was higher than those with no formal education (11.5%), those with high school education and vision impairment (10%) was lower than that for both primary education and no formal education. These results could have been influenced by the fact that 3% of the sample did not state their level of education although this group had 14.7% vision impairment. Alternatively, it could be that in South Africa, given the high levels of unemployment, a school leaving certificate may be of limited value in finding jobs, as preference is generally given to the unemployed university graduates and, as such, many people are likely to be in a similar situation to those with lesser school education.

The categories with the least expenditure and the most expenditure have the highest prevalence of vision impairment. If we take expenditure as a proxy for income and level of poverty this may be explained by the fact that those with the least income are less likely to access eye care services. Conversely those with the highest level of income are more likely to have jobs with a higher demand for vision tasks such as administration jobs and are more affected by the lack of vision correction or by vision impairment because of other causes.

Employed individuals and those with a higher family income have a higher prevalence of self-reported vision impairment. This could be a consequence of greater vision demands in work situations, which impacts subjectively on an individual as opposed to someone who does not have a great demand for vision. This would be particularly relevant in situations where near vision is demanded, such as for reading or sorting out products.

Two of the four provinces (Western Province and North West Province) which self-reported the highest level of vision impairment do not have optometrists employed in the public sector. These provinces are better resourced than some of the other provinces but for the poor very little affordable options exist when they cannot access public sector optometrists and affordable spectacles. Given that refractive error was found in an epidemiological study to be the major cause of vision impairment in South Africa (Naidoo *et al.* 2013:113), the lack of optometrists and public sector access to spectacles may explain this disparity.

The higher self-reported vision difficulty in smaller families could be related to the fact that smaller households are composed of pensioners whose children live elsewhere and, as such, the relative influence of age-related conditions such as cataracts and presbyopia on vision impairment is magnified. The study showed that there was a steady increase in self-reported vision impairment from age 40 years and onwards, a peak prevalence at around 60 years and a subsequent decline post age 60 years, which correlates with the onset of presbyopia (ages 35–40 years) and cataracts (ages 55–60 years). South Africa enjoys one of the highest cataract surgery rates in sub-Saharan Africa; despite this a significant backlog of services exists. The current cataract surgical services may therefore explain the post age 60 years decline.

Those with health needs self-reported the highest percentage of vision impairment compared to social, documentation and education needs. The lack of health services in general could be indicative of the lack of access to eye care services for affected individuals and communities. This could also be influenced by other co-morbidities such as HIV, diabetes and hypertension leading to a prioritisation of health care needs amongst respondents. Some of these conditions also impact vision.

### Limitations of the study

This study has limitations as the questions regarding vision difficulty were limited in the WOPC questionnaire. Further in-depth interviews and questions pertaining to poverty and vision difficulties will provide more information on the link between socio-economic status and vision problems. Unfortunately this was not possible at this stage, as the investigators analysed secondary data that were extracted from the WOPC questionnaire. A further limitation to the study was that vision difficulty in young children can be problematic to report as they are unable to effectively complain about an eye condition. In addition, it is difficult for parents to identify and report when young children have vision problems or diseases as many poor households do not access adequate health services to be able to detect vision problems early, unless they are severe or have been detected at birth.

## Conclusion

The results of this study serve as an indicator of people's perception of their vision status and difficulties experienced. The evidence from this study suggests associations between socio-economic factors and vision difficulties that have a two-fold relationship: some factors, such as access to eye health services may lead to vision difficulty whilst vision difficulty may trap people in their current poverty or deepen their poverty status. However, the responses of the subjects do not indicate a clear relationship between income and employment and vision difficulty in KwaZulu-Natal as income was scarcely reported by the subjects enumerated and there is a higher prevalence of vision difficulties amongst the employed individuals in comparison with those who indicated that they were unemployed. Despite this, it should be noted that many individuals were found to be employed in areas of work that are likely to require good vision and perform tasks that may have increased vision demands, which may present vision difficulties such as painting, bricklaying welding, plastering plumbing. However given the relationship between vision demands and a greater awareness of vision impairment in work situations, it is necessary to investigate further the unemployed group via clinical evaluations as well as comprehensive qualitative studies to determine if a vision difficulty restricts their capacity to gain employment. There are also other dimensions to poverty such as social well-being, health and living conditions, and others, which impact on vision but have not been investigated in this study in relation to vision difficulty. The current data are therefore insufficient to examine this relationship. The results should therefore be considered as indicative of further areas for research.

The authors have earlier presented specific research questions that need further investigation on poverty and eye health (Jaggernath *et al.* 2014:1855). Research areas that emanate from the intrinsic results and limitations from this study that are specific to the South African context, are highlighted in Table 3.

**TABLE 3:** Research areas emanating from the study.

Research area	Research interests
Income	Expenditure was utilised as a proxy to income in this study because the question pertaining to income in the WOPC questionnaire was scarcely reported. This could likely be because of the respondents not wanting to disclose their actual income to government as they may feel that they will be denied government grants if they state an income that is too high, or that they may be feeling a sense of inferiority and were afraid of the enumerator's perception of their response and thus avoided the question. However expenditure does not provide the actual earnings of the individuals in the households that were surveyed and would need to be cross-tabulated against household's income. Thus, a national study that does not place focus on poverty only, such as an investigation into poverty and eye health study, can help to elicit responses on income (in categories), without the fear of respondents being afraid to declare their earnings.
Inequality	Considering the high rates of unemployment, poverty and inequality in South Africa, especially within race and specifically on the African population, it is imperative that further studies be conducted to investigate the rate of inequality with regard to income, in the African population and within the different historical racial categories.Furthermore, despite advances in areas such as electrification and access to education that have increased equality of opportunities, more research is needed to determine what other opportunities (social, economic and health) have increased in terms of equality. Specifically, studies that investigate inequality in access to opportunities that improve eye health should be carried out.
Migration patterns	Leibbrandt *et al.* (2010) indicate that the urban population increased, thus causing an increase in the number of urban poor, and consequently a decrease in the number of rural poor people. The migration patterns need to be more closely studied to determine the push and pull factors for this migration pattern and the reasons for increased poverty in urban areas. Studies are therefore needed to investigate poverty in the urban areas and the subsequent, if any, impacts on eye health.
Eye health services	The study suggested that those with health needs have a higher percentage of vision impairment compared to other needs such as social, education and documentations. Lack of health services in general, could be indicative of the lack of access to eye care services for affected individuals and communities. An investigation needs to be conducted on the barriers that poor people face with regards to access to eye health services.
Gender and access to eye health services
Household priorities and eye health	In this study, household size was a significant factor in the self-reporting of vision impairment. An assumption of the authors are that larger houses mean that there are lesser resources or that limited resources for poor people need to be prioritised before eye health; thus vision problems are less of a priority and remain untreated. However, further investigation on the association between household size, household priorities and eye health need to be carried out to verify this assumption.
Vision difficulties in children
